# Neuroimaging and modulation in obesity and diabetes research: 10th anniversary meeting

**DOI:** 10.1038/s41366-021-01025-8

**Published:** 2021-12-21

**Authors:** Maren Laughlin, Bradley Cooke, Kerri Boutelle, Cary R. Savage, Alexxai Kravitz, Dana Small, Zoe Arvanitakis, Alex Martin, Luke E. Stoeckel

**Affiliations:** 1 NIH, National Institute of Diabetes, Digestive and Kidney Diseases, Bethesda, MD USA; 2 University of California, San Diego, CA USA; 3 University of Nebraska, Lincoln, NE USA; 4 Washington University in St. Louis, St Louis, MO USA; 5 Yale University, New Haven, CT USA; 6 Rush University, Chicago, IL USA; 7 National Institute of Mental Health, Bethesda, MD USA; 8 National Institute on Aging, Bethesda, MD USA

**Keywords:** Obesity, Type 2 diabetes

## Background

The National Institute of Diabetes and Digestive and Kidney Diseases (NIDDK) hosted a workshop on April 16–17, 2019 titled, “Neuroimaging and Modulation in Obesity and Diabetes Research: 10th Anniversary Meeting”. This workshop convened creative scientists conducting basic and applied obesity and type 2 diabetes (T2D) neuroimaging and neuromodulation research, and celebrated the substantial progress made in the decade since a similar previous NIDDK workshop entitled “Neuroimaging in Obesity Research” on October 27–28, 2008. The goals of the 2008 workshop were to bring together an interdisciplinary audience to explore the state-of-the-art in the various disciplines related to obesity research that have successfully used neuroimaging, to identify obesity-related research questions that lend themselves best to imaging approaches, and to discuss best imaging practices, available opportunities and resources, and barriers to progress. To stimulate research in this area, the NIH issued a Request for Applications, DK-08-009 “Neuroimaging in Obesity Research (R01)” http://grants.nih.gov/grants/guide/rfa-files/RFA-DK-08-009.html. For the 2019 workshop, attendees considered three questions (1) What have we done? (2) What are we missing? and (3) What can we do to move forward?

The World Health Organization estimates that the global incidence of overweight and obesity has *tripled* in the last 40 years [1]. Meanwhile, the number of people with T2D worldwide more than quadrupled, from 108 million in 1980 to more than 400 million in 2014 [2]. While the public health problem has been growing, researchers have focused on understanding the bidirectional relationships between obesity, T2D and brain structure and function, and how we can image and modulate the brain to advance our understanding of how it controls physiological and homeostatic functions as well as the cognitive and motivational states and traits critical for ingestive behaviors and metabolism [3].

The brain orchestrates what, when, and how much we eat and how our bodies use the nutrients provided by food. It drives our thirst and hunger, controls the sensory and emotional pleasures associated with eating and the decisions surrounding food choices. The complexity of feeding and metabolic circuitry could not have been imagined only 25 years ago, when the mediobasal hypothalamus was viewed as the “hunger center” and the lateral hypothalamus as the “satiety center” of the brain [4]. Now we know that feeding and metabolic control is mediated by discrete, genetically defined microcircuits within the mesoscopic anatomy of the brain [5]. The advent of neuroimaging technologies like functional magnetic resonance imaging (fMRI) has greatly improved our focus on the details of networks within the human brain [6].

The central control of metabolism involves hypothalamic signaling through the vagus nerve to the liver, fat and pancreas and the integration of reafferent signals in the brain stem and hypothalamus. These physiological signals are then projected to the anterior insula, which appears to regulate interoceptive attention to these signals before they are projected to higher-order brain networks that govern behavior [5]. These pathways are affected by obesity and T2D, which also lead to brain-peripheral system pathologies, impaired neuropsychological functions, and increased risk for Alzheimer’s disease and related disorders [7]. The 2019 workshop also addressed the advances that have enriched our understanding of these detailed networks and functions, which will help the community identify the gaps and research opportunities to be addressed over the next decade.

## Keynote addresses

### The neurobiology of homeostasis

Brains are highly complex in terms of scale, connectivity, and cell type diversity. NIH-led initiatives like the Brain Research through Advancing Innovative Neurotechnologies (BRAIN) Initiative are advancing our understanding by accelerating the development and application of innovative neurotechnologies. Examples include CLARITY, which removes optical barriers to mapping the physical connectivity of the brain [8] and optogenetics, which uses light-sensitive opsins in genetically identifiable cells to control neural activity while measuring behavior [9]. The keynote address by Dr. Zachary Knight (University of California—San Francisco, Howard Hughes Medical Institute) highlighted the power of neurotechnology to elucidate the basic functions of the brain.

The regulation of internal state, or homeostasis, is coordinated by specialized neural circuits that detect deviations in internal state and respond via compensatory physiological and/or behavioral mechanisms. Knight examined how we can better understand two key homeostatic functions relevant to obesity and T2D, thirst and hunger, by using optical tools to record the activity of the neuronal cell types involved. His talk first focused on neurons that control thirst, which are in the subfornical organ and median preoptic nucleus (MnPO). By monitoring the activity of these neurons when thirsty mice drink water, Knight showed that these cells receive three layers of signals that emerge sequentially during ingestion. The first set of signals arise from the mouth and throat, track drinking in real-time, and report on the volume of water ingested [10]. These oral cues are then followed a few minutes later by gastrointestinal signals that report on the osmolarity of ingested fluids [11]. Finally, fluid absorption into the blood, which occurs 10–15 min after the start of drinking, signals the restoration of systemic fluid balance. Knight showed that these three signals are seamlessly integrated by individual thirst-promoting neurons in the MnPO, allowing animals to dynamically track their need for water and rapidly adjust their drinking behavior [11].

Knight then drew parallels between this multi-level regulation of thirst circuits and his lab’s finding with respect to AgRP neurons that control hunger. By recording AgRP neuron activity in vivo, Knight showed that when a hungry mouse sees and smells food, these cells are inhibited within seconds in a way that is proportional to how many calories the mouse will eat in the subsequent meal [12]. He described how these neurons are then further inhibited on a timescale of minutes when the ingested nutrients are detected in the gut and on a timescale of hours when leptin levels rise due to increased fat mass [13, 14]. Knight suggested that this reveals a common logic for the regulation of hunger and thirst circuits, in which ingestion triggers layers of signals that emerge sequentially on timescales from seconds to minutes to hours that allow the brain to progressively update and refine its estimate of how much has been consumed [15].

Knight suggested several key takeaways and areas of importance for future research. First, he emphasized the idea that measurements of the in vivo dynamics of neural cell types should be the foundation for investigating brain function because only the natural dynamics of cell types can reveal what the cells do in a normal animal. He suggested that optogenetic manipulations are best used to test hypotheses generated by neural recordings, rather than as a first step to understand a neuron’s function and that the generation of “wiring diagrams” outlining the anatomy of neural circuits should be accompanied by investigation of how information is transformed from one node to the next. Finally, he proposed that the study of homeostasis would greatly benefit from new technologies for monitoring and manipulating cell types in peripheral tissues, such as the gastrointestinal tract, and highlighted recent advances along these lines that are already underway, such as his lab’s work mapping the vagus nerve [14].

### Toward precision neuroimaging in obesity and diabetes research

Precision medicine is an approach to disease treatment and prevention that takes into account individual variability in genes, environment, and lifestyle for each person rather than for the “average patient.” Dr. Russell Poldrack (Stanford University) described his vision for a “precision neuroscience” that would develop individually tailored treatments for neuropsychiatric conditions based on predictive modeling of person-specific behavioral, genetic, physiological, and neurophysiologic characteristics. Poldrack argued that to reach this “precision neuroscience” goal the field of neuroimaging must confront several major obstacles. These include the problems of irreproducible results, faulty or imprecise predictive analytic models, limited understanding of individual variability, understudied cultural and contextual factors in experimental design, and the challenge of addressing causal inference given current methodological limitations of neuroimaging tools.

Insufficient sample sizes and poorly understood statistical power in many clinical studies has led to problems with replication [16]. This problem is evident in the obesity fMRI literature where nearly 40% of recently published studies had sample sizes of 20 subjects or fewer (Poldrack, *unpublished*). Another problem is concerned with the reliability of the data itself. Although it has become increasingly common to use resting state scans to evaluate clinical group differences in functional connectivity, the amount of data collected from each individual subject is unlikely to provide a reliable estimate of network structure. In fact, whereas most fMRI studies collect 8–10 min of rest, an examination of the data collected from a single, highly sampled subject (84 sessions of ~9 min per session) indicated that 27 min of data (collected over three sessions) would be needed to achieve a split-half reliability of >90% [17, 18]. This would seem to be a nearly insurmountable financial and scanning time burden for most clinical studies—especially with the added need to include large numbers of subjects. However, this problem may be somewhat mitigated by noting that the same data indicates split-half reliability of >80% can be achieved with a single 9-min session. As recently shown in *Cell Reports*, 10 min of multiecho resting state data achieves better test-retest reliability than 30 min of typical (single echo) resting state data [19]. Thus, it now appears that advances in imaging methodology have made it likely that reliable resting state data can be obtained in considerably less time than initially thought.

Another obstacle to precision neuroscience is the limited data on how individual brain function varies over short to very long timescales. Thus, although we commonly assume that the functional organization of the adult brain is stable over years (aside from the effects of plasticity and aging) this assumption has not been directly evaluated. Interestingly, studies of single individuals suggest that dynamic changes in brain network structure may be related to lifestyle variables such as diet and mood [17, 20, 21]. The development of individually tailored treatments for obesity and T2D will depend on overcoming multiple obstacles, including imprecise predictive analytic models, limited understanding of individual variability, understudied cultural and contextual factors in experimental design, and the challenge of addressing causal inference given current methodological limitations of neuroimaging tools.

## Scientific sessions

### Session I: Setting the stage

Dr. Alain Dagher (McGill University) introduced the idea of the “omnivore’s dilemma” and drew from integrated neuroimaging, genetics, economics, sociology, and epidemiology data to support an “economic thrifty hypothesis” to explain the obesity epidemic. The hypothesis says that as the price of food decreases, food overconsumption is the adaptive and economically optimized response [22]. This would lead to an endophenotype that promotes over-consumption in the face of abundance. Big data analyses suggest that this endophenotype is best captured by a tendency for uncontrolled eating via reduced self-control and high food cue reactivity in the context of food surfeit [23]. One way such a phenotype could arise would be by less dorsolateral prefrontal control over the generation of ventromedial prefrontal value signals in response to food cues [24]. Another would be by strengthening excitatory signals from the cortex and hippocampus to the nucleus accumbens (NAc), which could promote food seeking and consummatory behaviors. These traits appear to be reflected in heritable brain morphometric findings measured by MRI [25]. Key takeaway points include 1) one phenotype for overeating includes high food cue reactivity and reduced self-control, which could be related to reduced prefrontal control over reward regions or activation of reward regions.

Non-human animal experiments support the idea that exposure to high calorie diets changes these circuits to favor over-consumption. Dr. Paul Kenny (Mount Sinai School of Medicine) reviewed data demonstrating that diet-induced obesity in male Wistar rats causes striatal dopamine D2 receptor down-regulation, which, in turn, supports compulsive responding for food [26]. He emphasized an often-overlooked observation from the study, which is that male Wistar rats given access to a high-fat/high-sugar diet for extended periods of time lost interest in consuming regular rat chow, even when the more palatable diet was unavailable. He also presented new data showing that a lesion to the hypothalamus-habenular circuit decreased motivation for chow and increased motivation for highly palatable food, while activation of this circuit had the opposite effect in normal weight animals (Kenny, *unpublished*). The lateral hypothalamus is thought to provide both excitatory and inhibitory input to the lateral habenula. In normal weight rats, chemogenetic inhibition of glutamatergic inputs from lateral hypothalamus to lateral habenula increases food intake, while inhibition of GABAergic inputs decreases food intake. The key takeaway point is that a new target for obesity intervention could be addressing the deficit in excitatory input to the habenula from the lateral hypothalamus.

Collectively, the two talks conveyed a single theme: that the decreased cost and increased availability of energy-dense palatable food increases consumption, which induces plasticity that biases the animal towards compulsive choices for high energy dense foods while simultaneously reducing the reinforcement value of healthier and lower energy-dense food.

### Session II: Imaging circuits involved in food intake and metabolic function (human studies)

Dr. Dana Small (Yale University) reviewed work using positron emission tomography (PET) to study dopamine signaling and its association with ingestive behavior and obesity. In her early work, she identified changes in binding potential (indicative of dopamine release) in the dorsal striatum following a favorite meal [27, 28]. She then reviewed reported results from PET studies looking at the association between binding potential and adiposity and found that reports have been inconsistent, with reports of increased binding potential [29–34], decreased binding potential [32, 35–38] or no association [39, 40] between binding potential for dopamine-linked radiotracers and measures of adiposity. Work in preclinical models [41–43] suggests that a high fat diet, independent of adiposity or metabolic dysfunction can influence dopamine circuits. Small, therefore, emphasized that inconsistent findings may reflect a failure to account for dietary influences [41, 44].

Dr. Eric Stice (Stanford University) continued the focus on the reward system of the brain, particularly the NAc, with his dynamic vulnerability model of obesity: Some individuals, such as those whose parents were obese, show elevated responsivity of reward regions to tastes of high-calorie foods, and are at risk for habitual intake of high-calorie foods. In turn, their habitual high-calorie food intake reinforces the hyper-responsivity of brain reward regions to high-calorie food cues, but surprisingly leads to *hypo*-responsivity to the tastes of these foods [45].

Stice and colleagues conducted a series of prospective, longitudinal studies to measure brain activity in lean individuals at risk for obesity. They found an elevated response to high-calorie food tastes relative to a low-risk group, but not to visual cues for expected high-calorie food tastes, consistent with the dynamic vulnerability model of obesity [46, 47]. Other studies found that elevated brain reward region response to high-calorie food images/cues was associated with higher BMI and predicted future weight gain, consistent with findings from natural history and weight-loss treatment studies [48–52]. He concluded his talk by providing evidence that a novel training intervention focused on food response inhibition and attention effectively reduces activation of reward and attention regions to high-calorie food images and led to decreased body fat [53], an effect whose replication trial is currently under review. Key takeaway point is prior to weight gain individuals at high risk to obesity have an elevated response to taste but not pictures which is associated with higher BMI and weight gain.

### Session III: Imaging circuits involved in food intake and metabolic function (non-human animal studies)

Rodent models have yielded many valuable insights over the past decade about the regulation of food intake and metabolic function. In this session, Dr. Carrie Ferrario (University of Michigan) presented data consistent with the hypothesis that the propensity to diet-induced weight gain is due in part to increased responsiveness to the sensory cues of food. Her lab collected behavioral data and electrophysiological recordings from rats bred to be susceptible or resistant to diet-induced obesity. As predicted, motivational responses to food cues were stronger in the obesity-susceptible rats before they became obese; moreover, Dr. Ferrario showed this is linked to increases in calcium-permeable AMPA receptors in the NAc [54–56], and other differences in NAc function in obesity-susceptible vs resistant groups [57, 58]. Key takeaway points include that enhanced neural and behavioral responsivity to food cues likely contribute to initial weight gain, as well as to the difficulty some have in maintaining weight loss [56, 59, 60].

Dr. Matt Carter (Williams College) presented data on an under-studied brain area known as the parasubthalamic nucleus (PSTN), which has a potential role in appetite regulation [61–63]. To test its role, he performed a genomic analysis of the PSTN, and determined that there are two populations of neurons in this region, one expressing *Tac1* and another expressing corticotrophin releasing hormone (CRH). A large meal and various anorexigenic hormones (Extendin-4, amylin, CCK) led to selective activation of the *Tac1* neurons, as measured by the early gene product Fos and real-time population calcium recordings. This suggests that Tac1 neurons play a role in satiety. Consistent with this, optogenetic and chemogenetic stimulation of the Tac1 neurons suppressed food intake, while CRH neurons were ineffective. Chemogenetic inhibition of Tac1 neurons, but not CRH neurons, attenuated the effects of anorexigenic hormones. Key takeaway points include that Tac1 and CRH neurons showed different projection patterns throughout the brain, with Tac1 neurons projecting to neural populations known to suppress food consumption.

### Session IV: Modulation approaches with potential for obesity and diabetes

In her talk, Dr. Polina Anikeeva (Massachusetts Institute of Technology) described how optic fiber-based and nanomagnetic tools can address longstanding as well as new questions. One of the more promising recent advances is in minimally invasive (i.e., implant-free) neuromodulation with magnetic nanoparticles to activate heat-sensitive transient receptor potential ion channels in mice. Dr. Anikeeva demonstrated how these technologies could be used to map projections from the ventral tegmental area, a key region in the brain’s reward circuitry, at an unprecedented level of precision and scale [64, 65]. However, she noted that there may be limits to the utility for engineering magnetically sensitive cells [66]. Using non-magnetic iron oxide (FeO) nanoparticles as controls for magnetic iron oxide (Fe_3_O_4_), she found that iron alone does not make things magnetic—it has the crystal structure that is important, suggesting new strategies and approaches for magnetothermal deep organ stimulation as means to study organ function and pave the way to development of bioelectronic medicines [67]. A key takeaway is that new materials technologies will increase the precision of non-invasive neuromodulation in vivo in nonhuman animals and there is early work showing magnetic nanoparticles may also be used as a potential MRI contrast theranostic (therapeutic and diagnostic) agent in humans.

Dr. Miguel Alonso-Alonso (Beth Israel Deaconess Medical Center and Harvard Medical School) reviewed existing therapeutic options for obesity, such as lifestyle modification and pharmacotherapy, and then focused on the potential of neuromodulation, such as transcranial magnetic or transcranial direct current stimulation (TMS or tDCS, respectively) [60]. With increasingly sophisticated human functional neuroimaging, our understanding of the neuroscience of obesity has broadened from a hypothalamus-centered perspective to a more whole-brain neurocognitive model. Consequently, new ideas, technologies, approaches, and neural targets have emerged [6]. For example, executive functions mediated by the dorsolateral prefrontal cortex have emerged as promising new targets for intervention via neuromodulation [6]. Invasive, deep brain stimulation and non-invasive TMS and tDCS are all being explored as treatments for obesity and T2D [68, 69]. Before we can realize the potential of these technologies, however, the field must address the rigor and reproducibility of the research in this area [70]. Key takeaway points include suggestions that TMS, tDCS and neuromodulation may offer promise for interventions for individuals with obesity. In addition, there has been much more limited work in this area related to T2D, but there is some evidence of impaired neuroplasticity associated with glutamatergic metabolite levels in prediabetes, as investigated by TMS and MR spectroscopy [71].

Dr. Casey Halpern (Stanford University) gave a provocative talk on a potential role for deep brain stimulation (DBS) in the treatment of obesity [72]. Halpern stressed the importance of focusing on individuals with obesity driven by loss of control of eating, since they are more likely to have an abnormality in a well-defined neural circuit involving inhibitory control. He highlighted the development of closed-loop or responsive DBS stimulation, in which patterns of neural activity that predict the loss of eating control leads to stimulation of key brain sites. For example, specific patterns of activity in the NAc occur when mice and humans are anticipating a reward. This activity pattern could, in principle, be used by a closed-loop system to suppress loss of control eating [73]. The next step is to determine whether this would be feasible for use in highly treatment-refractory individuals with obesity, which will be tested in an ongoing first-in-human trial (NCT03868670; [74]. A key takeaway point is that responsive DBS stimulation may offer promise for interventions for individuals with obesity.

### Session V: Imaging gut–brain interactions

One of the most important recent discoveries about ingestive behavior is the critical importance of the gut-brain axis in regulating food reward. Dr. Ivan de Araujo (Mount Sinai School of Medicine) presented work using cell-specific transneuronal tracing to reveal a functionally asymmetric ascending pathway originating from lipid sensing cells in the upper intestine and projecting via the vagus nerve through the right nodose ganglion to the nucleus of the solitary tract (NTS). Critically, he showed that mice will work to obtain optical stimulation of NTS neurons that receive right nodose ganglion inputs. NTS neurons, in turn, project to the parabrachial nucleus, substantia nigra, and finally the dorsal striatum where they regulate dopamine release, reward and reinforcement [75]. The finding has broad ranging implications. It not only establishes a neural gut-to-brain pathway that is integral to food reward, but it also suggests a link between diet-induced adaptations of this pathway and dopamine-dependent functions, such as affect, impulsivity and executive control.

Dr. Sarah Stanley (Mount Sinai School of Medicine) described an exciting non-invasive technology she developed to study the central regulation of glucose control. Her group was able to control hypothalamic glucose sensing neurons in vivo using transgenic mice and an ion channel linked to a ferritin molecule, allowing for non-invasive electromagnetic control of peripheral glucose metabolism and food intake in the mice. The advantage of this approach for basic studies is clear: unlike optical or electrical stimulation, this does not require a permanent implant. Several groups have applied similar magnetogenetic technologies to diverse studies; to regulate cell migration in vitro, determine the mechanisms underlying fever-associated birth defects and manipulate reward circuits in vivo [76–78]. Recent work identified local signaling by reactive oxygen species and oxidized lipids as key mechanisms for electromagnetic control of temperature sensitive channels [79, 80]. These findings will guide the development of next generation magnetogenetic tools. A key takeaway point is this novel tool for rapid, non-invasive modulation of specific neural circuits in freely moving animals holds considerable promise for understanding the neural control of metabolism [81].

### Session VI: Mechanisms of neurocognitive dysfunction in obesity and diabetes

Dr. Ellen Schur (University of Washington) discussed the role of hypothalamic gliosis (i.e., neuroinflammation) in rodent models of diet-induced obesity, arguing that glial cell inflammatory responses are necessary and sufficient for hyperphagia and weight gain [82–84]. She also introduced quantitative T2 MRI as a useful, non-invasive tool for investigating hypothalamic gliosis in humans in vivo. Early converging evidence suggests that hypothalamic gliosis can be detected in humans and that this may be a contributing factor to obesity and T2D [85]. Key unanswered questions in humans include whether gliosis is reversible, whether there are dietary triggers for gliosis, and how gliosis may influence eating behavior. Key takeaway points include inflammatory responses in glial cells are associated with weight gain and can be measured in humans using T2 MRI.

Dr. Scott Kanoski (University of Southern California) explained how excessive consumption of simple sugars during juvenile-adolescence in rodents disrupts aspects of hippocampus-dependent learning and memory. This appears to be specific to early development as Kanoski did not observe disrupted hippocampal function following 30 days of adult consumption of simple sugars [86]. Moreover, the memory impairment associated with early life excessive sugar consumption may not be reversible as normalization of hippocampal function was not observed months after removal of simple sugars from the diet [87]. Recent findings from the Kanoski lab support an association of simple sugar consumption on hippocampal function with alterations in the gut microbiome. Specifically, early life excess sugar consumption in rats increases the abundance of bacteria in the genus *Parabacteroides* that are negatively correlated with hippocampal function; targeted enrichment of *Parabacteroides* in the gut microbiome impairs hippocampal-dependent memory in the absence of sugar consumption [88]. The key takeaway point is that early exposure to sugar impairs hippocampal dependent memory through changes in the gut microbiome which may not be reversible.

Dr. Alexis Stranahan (Medical College of Georgia) discussed the differences between brown adipose tissue, visceral white adipose, and subcutaneous white adipose and their potential role in obesity-associated cognitive impairment. She presented data in rodents demonstrating that obesity leads to increased blood–brain barrier (BBB) permeability and macrophage infiltration, and that visceral adiposity specifically increases levels of the cytokine interleukin 1b, promotes microglial activation (an inflammatory response), and induces hippocampal dysfunction [89]. Her group also identified a role for endothelial adenosine receptor 2A activation in obesity-induced BBB breakdown and cognitive impairment [90]. A key takeaway point is that different fat depots may adversely impact the brain via different mechanisms with some types of fat more detrimental to the brain (e.g., visceral adiposity promoting microglial activation and inducing hippocampal dysfunction).

### Session VII: Developmental considerations in obesity and diabetes

Drs. Sean Deoni (Brown University) and Susan Carnell (Johns Hopkins University) discussed children’s brain development in the context of prenatal maternal and early childhood obesity and their links to cognitive impairments and alterations in brain structure and function. Deoni and Carnell described the Resonance study, a large-scale longitudinal brain imaging cohort beginning in infancy and focusing on neurodevelopment and broad environmental exposures. They shared preliminary resting state fMRI data showing that maternal pre-pregnancy obesity is associated with increased intra-hemisphere and decreased inter-hemisphere connectivity, suggesting altered trajectories of early brain development. Dr. Carnell then described the biobehavioral susceptibility model of obesity, which proposes that genes (e.g., *FTO*, fat mass and obesity-associated) and family environment interact to influence appetitive traits, including the responsivity to food cues in the wider environment [91, 92]. She described parent-report questionnaires, e.g., Child Eating Behaviour Questionnaire; Baby Eating Behavior Questionnaire, [93, 94] used to assess appetitive behavior in infants and children, and relationships between *FTO*, appetitive behavior and body weight. She showed that familial obesity risk as reflected by maternal overweight is associated with lower activation to food cues in a self-regulation circuit including the dorsolateral prefrontal cortex, and that food approach behaviors are associated with activation of regions involved in sensing or anticipating reward, including the insular cortex. Subsequent work has further revealed reduced gray matter volume and cortical thickness in regions subserving gustatory processing and self-regulation in association with familial obesity risk [95]. Key takeaway points include (1) maternal pre-pregnancy obesity impacts connectivity within and between hemispheres and (2) maternal obesity influences the child’s neural self-regulation and reward circuits in response to food cues, as well as regions focused on gustatory processing.

Dr. Katie Page (University of Southern California) reviewed the role of human hypothalamus in the regulation of glucose metabolism and appetite. She demonstrated effects of table sugar *versus* non-nutritive sweeteners on brain and hormonal function and their roles in appetite, eating behavior, and obesity risk [77]. She found that exposure to mother’s diabetes is associated with altered function in the child’s hypothalamus. These hypothalamic abnormalities were associated with greater future increases in children’s BMI [96]. Her group recently showed that prenatal exposure to gestational diabetes is also associated with greater food-cue elicited activity in the orbitofrontal cortex and greater daily energy intake, which in turn was associated with greater abdominal adiposity in children [97]. These findings, along with those of Deoni and Carnell, show that risk of obesity is profoundly affected by genetic and environmental forces acting well before birth, which has important implications for prevention and treatment development efforts.

## Conclusions and future directions

A major theme that emerged from the workshop is that *obesity begets obesity* (and, by extension, metabolic disease, i.e., T2D begets T2D). Obesity and diabetes can begin in the womb, driven by a complex interplay of genes, behaviors, and environment [91, 92, 95–97]. Brain imaging is helping to elucidate how genes and epigenetic marks influence body weight directly by controlling development of hypothalamic and other brain circuitry, and that of the rest of the body such as the visceral organs. Heritable traits are also passed on indirectly, transmitting the consummatory behavior of the mother to the fetus via the placenta and then to the child through the mother’s food choices. It is likely, therefore, that the combination of traits leading to obesity are highly personal. This fact about human development is underscored by the emphasis on precision medicine. As highlighted by President Barack Obama, precision medicine aims to “deliver the right treatments, at the right time, to the right person every time” (*All of Us*). The workshop also highlighted advances in tools, methods, and computational approaches building towards a precise neuroscience-driven approach to the prevention and treatment of obesity and T2D.

Of course, a great deal of work remains to be done. In addition to furthering our understanding of the central regulation of feeding behavior and metabolic control, more research is needed to elucidate the descending and reafferent signals of peripheral metabolic tissues. Another layer of complexity derives from the observation that brain physiology and function are highly responsive to the status of the body, including diet and obesity. Drs. Small and Kenny reported that a high fat diet blunts dopamine signaling and shifts preference from low to higher fat foods, even in the absence of weight gain. Dr. Kanoski demonstrated impaired hippocampal dependent learning and memory in rodents following a diet high in sugar, and Dr. Page showed distinct effects of caloric and non-caloric sweeteners on hypothalamic responses and reported that maternal diabetes is associated with altered hypothalamic function in children that is associated with weight gain. Dr. Stice’s dynamic vulnerability model posits that elevated brain reward responses to high-calorie food tastes prompts escalated intake of these foods, which results in hyper-responsivity of brain reward regions to cues for high-calorie food availability, and *hypo*-responsivity to the tastes of these foods. This results in eating in the absence of hunger. Collectively, these data point to a role for an interaction between the food environment and brain function that drives overeating and obesity and results in metabolic dysfunction, consistent with Dr. Carnell’s biobehavioral susceptibility model of obesity beginning in childhood.

Optical tools with larger and deeper fields of view, better spatial and temporal resolution, and novel imaging agents will allow behavioral neuroscientists to more fully describe the interacting biological systems that control energy status, while computational/algorithmic frameworks are needed to reduce that complexity to describe how information flows and is transformed by the brain. Progress will come not only from finer distinctions among the brain’s neural and glial constituents, but also from greater holistic integration across the entire brain, so that the causal dynamics during feeding and metabolic control can be observed. Imaging data could inform a “metabolic brain connectome” of neural connections and neurotransmitter signals in the brain that regulate energy balance and metabolism. The ongoing NIH BRAIN Initiative may provide powerful tools and methods, and new fields and communities have emerged to leverage advances in computational science and artificial intelligence.

Ultimately, data collected via imaging should inform treatment. Despite their efficacy for some psychiatric disorders, current neuromodulation technologies have less utility for obesity, although clinicians are working to more effectively target deep structures [98]. Development of technologies to target the peripheral nervous system, particularly the vagus nerve, have been hindered by the mix of sensory and motor axons and the myriad targets of those fibers, which make it difficult to reliably stimulate axons that specifically innervate the liver or pancreas. Decoding the signals of the vagus nerve will improve clinicians’ ability to identify and target specific axons. Behavioral therapies, such as reduction of food valuation via diet and food attention/response training paradigms are also informed by imaging studies.

Progress in this field is intimately dependent on engagement with and among people. The workshop highlighted the value of collaborations between experts in animal models, clinical researchers, and the creative scientists who develop tools and technologies. Psychologists, physicians focused on obesity and metabolic diseases, biologists interested in eating behavior and energy balance, engineers, imaging experts and computational scientists are all needed, and these teams are reliant on interactive environments where multi-disciplinary teams can thrive. The participants studied in clinical research are equal partners, important for the development of precision medicine approaches to combat obesity. Researchers should endeavor to include diverse populations most affected by these chronic health conditions and work to advance health equity. Disentangling the many neural substrates of obesity and T2D must include the contribution of social determinants of health to complete the picture of how heredity and experience interact to shape a person’s phenotype, setting the stage for precision therapies.
