# Correction to: Neuroimaging and modulation in obesity and diabetes research: 10th anniversary meeting

**DOI:** 10.1038/s41366-022-01074-7

**Published:** 2022-01-19

**Authors:** Maren Laughlin, Bradley Cooke, Kerri Boutelle, Cary R. Savage, Alexxai Kravitz, Dana Small, Zoe Arvanitakis, Alex Martin, Luke E. Stoeckel

**Affiliations:** 1 NIH, National Institute of Diabetes, Digestive and Kidney Diseases, Bethesda, MD USA; 2 University of California, San Diego, CA USA; 3 University of Nebraska, Lincoln, NE USA; 4 Washington University in St. Louis, St Louis, MO USA; 5 Yale University, New Haven, CT USA; 6 Rush University, Chicago, IL USA; 7 National Institute of Mental Health, Bethesda, MD USA; 8 National Institute on Aging, Bethesda, MD USA

**Keywords:** Obesity, Type 2 diabetes

Correction to: *Int J Obes* 10.1038/s41366-021-01025-8

The original version of this article unfortunately contained a mistake. The following statement was missing in the article: Disclaimer Statement: The content is solely the responsibility of the authors and does not represent the official views of the NIH or federal government.

Acknowledgements

The workshop entitled “Neuroimaging and Modulation in Obesity and Diabetes Research 10th Anniversary Meeting”, that served as an important foundation for this review article, was supported by the NIDDK of the NIH. We would like to thank the authors for their service on the workshop planning committee. BC, ML, and LES were lead authors. All other listed authors contributed equally to the workshop report. All listed speakers contributed to authorship for their section of the report.

The authors apologize for the mistake. The original article has been corrected.

